# Treatment and outcomes of mechanical complications of acute myocardial infarction during the Covid-19 era: A comparison with the pre-Covid-19 period. A systematic review and meta-analysis

**DOI:** 10.1515/med-2022-0545

**Published:** 2022-09-06

**Authors:** Cristiano Spadaccio, Angelo Pisani, Antonio Salsano, Antonio Nenna, Alexander Fardman, David D’Alessandro, Francesco Santini, Mario F. L. Gaudino, Thoralf M. Sundt, David Rose

**Affiliations:** Cardiac Surgery, Massachusetts General Hospital (MGH), Harvard Medical School, Boston, United States of America; Cardiac Surgery, Pineta Grande Hospital, Castel Volturno, Naples, Italy; Division of Cardiac Surgery, IRCCS Ospedale Policlinico San Martino, Genoa, Italy; DISC Department, University of Genoa, L.go Rosanna Benzi, 10, 16143, Genoa, Italy; Division of Cardiac Surgery, Università Campus Bio-Medico di Roma, Rome, Italy; Lev Leviev Heart and Vascular Center, Sheba Medical Center, Tel Hashomer, Israel; Sackler School of Medicine, Tel Aviv University, Tel Aviv, Israel; Cardiothoracic Surgery, Weill Cornell Medicine, New York, United States; Cardiac Surgery, Lancashire Cardiac Centre, Blackpool Victoria Hospital, Blackpool, United Kingdom

**Keywords:** myocardial infarction, mechanical complications, Covid-19

## Abstract

This study aims to compare treatments and outcomes of mechanical complications of acute myocardial infarction (MI) during the Covid-19 and in the pre-Covid-19 era. Electronic databases have been searched for MI mechanical complications during the Covid-19 era and in the previous period from January 1998 to January 2020 (pre-Covid-19 era), until October 2021. To perform a quantitative analysis of non-comparative series, a meta-analysis of proportion has been conducted. Early mortality after surgical treatment was 15.0% while it was significantly higher after conservative treatment (62.4%) (*P* = 0.026). Early mortality after surgical treatment was seemingly higher in the pre-Covid-19 era but the difference did not reach statistical significance (15.0% vs 38.9%; *P* = 0.13). Mortality in patients treated conservatively, or turned down for surgery, was lower during the Covid-19 pandemic (62.4% vs 97.7%; *P* = 0.001). The crude mean prevalence of the use rate of conservative or surgical treatment across the studies during Covid-19 and in the pre-Covid-19 era was comparable. The current increased incidence of MI mechanical complications might be a consequence of delayed presentation or restricted access to hospital facilities. Despite the general negative impact of Covid-19 on cardiac surgery volumes and outcomes and the apparent increase of the incidence of MI complications, the outcomes of their surgical and clinical treatment seem not to have been affected during the pandemic.

## 1 Introduction

Covid-19 pandemic has determined an unprecedented burden on the delivery of cardiovascular care worldwide [1,2,3,4]. An increasing amount of reports have been published on the development of mechanical complications of acute MI [5] as a direct consequence of the delayed presentation and inability to receive adequate treatment [6,7,8,9]. We performed a metanalysis of the currently available data on MI mechanical complications with the aim to describe the epidemiological characteristics of this phenomenon and compare it with the temporal trends and outcomes of the pre-Covid-19 era.

## 2 Methods

Study design is available online (PROSPERO registration CRD42021276091), and database search is updated to the end of October 2021. Full details are included in Supplemental Material. This study complies with the Declaration of Helsinki. Local ethics committee approval was not required considering the study design (secondary research not directly dealing with human subjects).

### 2.1 Search strategy

MEDLINE and the Cochrane Library databases have been systematically. Search strategies including exploded MeSH terms have been used. Search strings have been reported as Supplementary Material. English language restriction was imposed. Additional articles by manually searching the reference lists from recent reviews and the extracted papers have been looked for. Attempts have been made at collecting unpublished data from the authors of potentially pertinent papers.

### 2.2 Study selection criteria

Letters, editorial, reviews, animal studies, and reports with duplication data have been excluded. PICOS study design was used for inclusion/exclusion criteria. To identify eligible studies, a two-step selection process has been applied. Three reviewers (AS, CS, and AN) checked the eligibility criteria and selected the studies for inclusion in the present systematic review. Three researchers (AS, CS, and AN) independently screened records for inclusion. They were blinded to each other’s decisions. Disagreements between individual judgements have been resolved by consensus. Studies were excluded if they did not meet the criteria.

### 2.3 Data extraction and quality assessment

Three investigators (AS, CS, and AN) independently extracted data from all eligible studies using a standardized Excel file, focusing on study design, study size, type of intervention, and outcomes. Any disagreement was solved by consensus. We assessed the study quality with the Newcastle–Ottawa quality assessment scale.

### 2.4 Statistical analysis

To perform a quantitative analysis of non-comparative series, a meta-analysis of proportion has been conducted. To draw statistical inferences from heterogeneous studies, we employed non-iterative estimate of the inter-study variance component based on a random effects model (s2), taking into account that statistical heterogeneity is believed to be due to clinical diversity. Tau^2^ = 0 indicates no between-study heterogeneity. Double arcsine transformations have been applied to the observed proportions identified across a collection of studies to make the transformed proportions follow a normal distribution to accurately estimate the summary proportion and increase the validity of the associated statistical analyses. Multiple meta-regression with the Knapp–Hartung adjustment has been used to test the influence of the publication date and Covid-19 on early mortality. A *P* value of 0.05 was considered statistically significant. Statistical analyses have been done using the packages “meta” and “metafor” of *R* software, version 4.0.5.


**Ethic statement:** This study complies with the Declaration of Helsinki. Reviews, meta-analyses, or descriptions of educational materials do not involve human subjects and do not require IRB review (Grad Med Educ. 2011 Mar; 3(1): 5–6. doi:10.4300/JGME-D-11-00005.1). This study does not directly involve human participants.
**Patient and public involvement:** Patients or the public were not involved in the design, or conduct, or reporting, or dissemination plans of our research

## 3 Results

The pooled prevalence of early mortality independently on the treatment received during the Covid-19 pandemic was 31.1% (95% CI 12.7−52.0%, pooled data from 67 patients in 30 studies including case reports, case series, and observational studies). When compared to patients treated before the Covid-19 pandemic (pooled data from 8,647 patients in 47 studies including case series and observational studies), there was no difference in the overall early mortality (independently on the treatment received) (46.4% vs 31.1%; *P* = 0.24). Early mortality after surgical treatment was seemingly higher in the pre-Covid-19 era, but the difference did not reach statistical significance (*P* = 0.135). Conversely, mortality in patients treated conservatively, or turned down for surgery, was lower during the Covid-19 pandemic (*P* = 0.001). The crude mean prevalence of the use-rate of conservative or surgical treatment across the studies during Covid-19 and in the pre-Covid-19 era was comparable, when considering only the studies reporting both the approaches (surgery: 56.7% vs 66.3%; conservative 43.3% vs 33.7%; *P* = 0.13). After adjusting for the publication date, the period relative to Covid-19 pandemic seemed to influence the effect size for early mortality after conservative treatment but did not have impact in the surgical group. Results are summarized in Table 1 and described in detail in Supplemental Material.

**Table 1:** Treatments and outcomes of mechanical complications of MI considering Covid-19 pandemic

Results of the metanalysis	Pooled results	Treatments/timing		*P* value	Egger’s test
Early mortality during Covid-19 pandemic, surgery vs no surgery	67 patients	Surgery	No surgery	*P* = 0.026	*P* = 0.38
	31% (13–52%)	22 studies, 38 patients	13 studies, 29 patients		
	*I* ^2^ = 26%	15% (1–37%), *I* ^2^ = 9%	62% (27–93%), *I* ^2^ = 35%		
Early mortality after surgery, before vs during Covid-19 pandemic	8,504 patients	Surgery before Covid-19	Surgery during Covid-19	*P* = 0.135	*P* = 0.77
	35% (31–40%)	47 studies, 8,466 patients	22 studies, 38 patients		
	*I* ^2^ = 87%	39% (35–43%), *I* ^2^ = 91%	15% (1–37%), *I* ^2^ = 9%		
Early mortality after conservative treatment, before vs during Covid-19 pandemic	210 patients	No surgery before Covid-19	No surgery during Covid-19	*P* = 0.001	*P* = 0.06
	95% (80–100%)	8 studies, 181 patients	13 studies, 29 patients		
	*I* ^2^ = 67%	98% (94–100%), *I* ^2^ = 0%	62% (27–93%), *I* ^2^ = 35%		

Results of the meta-regression	Coefficient	Standard error	95% Confidence interval	*P* value	Interaction
Surgery					
Year of publication	<0.01	0.003	−0.006/0.006	*P* = 0.982	*P* = 0.88
Covid-19 pandemic	−0.12	0.090	−0.300/0.058	*P* = 0.185	
Conservative treatment					
Year of publication	<0.01	0.009	−0.019/0.020	*P* = 0.964	*P* = 0.56
Covid-19 pandemic	−0.53	0.140	−0.820/−0.250	*P* = 0.001	

To perform a quantitative analysis of non-comparative series, a meta-analysis of proportion has been conducted. To draw statistical inferences from heterogeneous studies, we employed non-iterative estimate of the inter-study variance component based on a random effects model, considering that statistical heterogeneity is believed to be due to clinical diversity. Double arcsine transformations have been applied to the observed proportions identified across a collection of studies to make the transformed proportions follow a normal distribution. Multiple meta-regression with the Knapp–Hartung adjustment has been used to test the influence of the publication date and Covid-19 on early mortality.

## 4 Discussion

Mortality for MI mechanical complications during Covid-19 pandemic was not dissimilar from the pre-Covid-19 era. However, underreporting of submersed populations of patients not reaching medical attention because of the gravity of the disease and/or the restricted access to specialized hospital facilities could have clearly affected our results. In support of this hypothesis, an increase in the incidence of out-of-hospital cardiac arrests and in the number of patients declared deceased on scene has been demonstrated in areas at high Covid prevalence [10,11].

Despite many reports advocated a worrisome disruption of the cardiac surgical and interventional activities due to the unprecedented overload of the healthcare facilities [2,3,6,8,12], the relative rate of surgical procedures for MI complications and surgical turndowns to conservative management did not change in respect to the pre-Covid-19 era. This might suggest that notwithstanding the significant resource and logistic burden posed on cardiac units, the ability to deliver high standards of care in these high-risk cases was not compromised during the pandemic.

Reasons underlying the results of surgery for MI complications and the apparent improved outcomes of conservative management during Covid-19 pandemic cannot be inferred with the present analysis. The modern improvements of intensive care and medical management might have played a role in the outcomes of the conservative subgroup, as suggested by the meta-regression analysis. However, significant selection and publication biases, implying a large population of critically ill patients not reaching medical attention, or a relatively more stable subset of patients being treated and reported during the pandemic, impedes to establish any explanatory or causative link in this context. However, it is intriguing to note that in the general impetus for publication during the Covid-19 era, there is a seemingly more frequent report of conservative treatments in the surgical literature. Whether this finding suggests that surgery is less offered in the recent era is difficult to ascertain, but it could at least partially balance the publication selection bias typical of the pre-Covid-19 periods. However, as this analysis is comparing consecutive rather than contemporary cohorts, the impact of the progressive advancements in the practice across the years in all the fields of medicine should also be considered. As additional limitations of this study, it remains difficult to evaluate if emergency cardiac surgical services have returned to normal in each country, and therefore, the “Covid era” has a different time span in each country. Moreover, there are no data about the percentage of patients with mechanical complications being offered or receiving surgery to be compared with the percentage of patients declined or turned down for surgery, in both eras; these data were not derivable from published studies and a possible “cherry picking” of low-risk cases by surgeons cannot be disproved.

## 5 Conclusions

Besides the inherent limitations of this study, this meta-analysis first and comprehensively described the current evidence on the management of MI complications during the pandemic. Despite the general negative impact of Covid-19 on cardiac surgery volumes and outcomes and the apparent increase of the incidence of MI complications, the outcomes of their surgical and clinical treatment seem not to have been affected during the pandemic.

## Supplementary Material

Supplementary Table
