# Supplementary material for: Treatment and outcomes of mechanical complications of acute myocardial infarction during the Covid-19 era: A comparison with the pre-Covid-19 period. A systematic review and meta-analysis
Source: Open Med (Wars). 2022 Sep 6;17(1):1412–6. doi: 10.1515/med-2022-0545 (PMC9449689; doi:10.1515/med-2022-0545)
Supplement: Supplementary Table [file med-2022-0545-sm.pdf]

SUPPLEMENTAL MATERIAL

Treatment and outcomes of mechanical complications of acute myocardial infarction during the Covid-19 era: a comparison with the pre-Covid-19 period. A systematic review and meta-analysis.

Study question and outcome's definition. Literature search strategy. 2
Methods. 3
PICOS criteria for inclusion and exclusion of studies into meta-analysis. 4
Quality assessment of the included studies. 5
Characteristics of the included studies. 7
Supplementary References. 8
Flow chart of search strategy. 11
Pooled prevalence of early mortality during Covid-19 pandemic. 12
Funnel plot and radial plot for early mortality during Covid-19 pandemic. 13
Leave one out forest plot for early mortality during Covid-19 pandemic. 14
Pooled prevalence of early mortality after surgery before and during Covid-19 pandemic. 15
Funnel plot and radial plot for early mortality after surgery before and during Covid-19 pandemic. 16
Pooled prevalence of early mortality after conservative treatment before and during Covid-19 pandemic. 17
Funnel plot and radial plot for early mortality after conservative treatment before and during Covid-19 pandemic. 18
Results of multiple meta-regression analysis for early mortality 19
PRISMA checklist 20

This supplementary material has been provided by the authors to give readers additional information about their work.

Study question and outcome's definition

Study question: Has the covid pandemic affected the management and outcome of patients with mechanical complications of acute myocardial infarction?

Main outcome. Early mortality: in-hospital/30 day mortality.

Literature search strategy.
Searched information sources listed (ie, PubMed, Cochrane database)

- Terms used for electronic literature search provided
- Reasonable limitations placed on search (ie, English language)
- Manual search conducted through references of articles, abstracts
- Attempts made at collecting unpublished data

Our keywords and MeSH terms pertinent to the exposure of interest were used in relevant combinations and they are showed below.

**Website**	https://www.ncbi.nlm.nih.gov/pubmed<br>https://www.scopus.com/
**Access**	June, 2021
**Filters**	English language
**Fields**	Title, Abstract
**Search terms**	**"**mechanical complications" and ("acute myocardial infarction" or "ST-elevation myocardial infarction" or "STEMI")

Additional search manual search conducted through references of extracted articles; "acute mitral regurgitation" and "myocardial infarction" and "surgery" OR "mitral valve repair" as additional string search term; attempts made at collecting unpublished data from the Authors of potentially pertinent papers

Number of articles: 100

Methods:

A meta-analysis of observational studies examined early mortality of patients with mechanical complications due to acute myocardial infarction.
This review has been registrered in PROSPERO (https://www.crd.york.ac.uk/PROSPERO) with the following registration number: CRD42021276091.

Search Strategy
MEDLINE and the Cochrane Library databases have been systematically. Search strategies including exploded MeSH terms have been used. Search strings have been reported as supplementary material. English language restriction was imposed. Additional articles by manually searching the reference lists from recent reviews and the extracted papers have been looked for. Attempts has been made at collecting unpublished data from the Authors of potentially pertinent papers.

Study Selection Criteria
Letters, editorial, reviews, animal studies and reports with duplication data have been excluded. PICOS study design was used for inclusion/exclusion criteria (Supplemental material). To identify eligible studies, a two-step selection process has been applied. 3 reviewers (AS, CS and AN) checked eligibility criteria and selected the studies for inclusion in the present systematic review. 3 researchers (AS, CS and AN) independently screened records for inclusion. They were blinded to each others' decisions. Disagreements between individual judgements have been resolved by consensus. Studies were excluded if they did not meet the criteria.

Data Extraction and Quality Assessment
Three investigators (AS, CS and AN) independently extracted data from all eligible studies using a standardized Excel file, focusing on study design, study size, type of intervention and outcomes. Any disagreement was solved by consensus. List of excluded studies with explained reasons were detailed in Supplementary Table 2. We assessed the study quality with the Newcastle–Ottawa quality assessment scale (NOS) [Wells GA, Shea B, O'Connell D, et al. The Newcastle-Ottawa Scale (NOS) for assessing the quality of nonrandomised studies in meta analyses. http://www.ohri.ca/programs/clinical_epidemio logy/oxford.asp. Accessed 15 June 2012.].

Statistical Methods
To perform a quantitative analysis of non-comparative series, a meta-analysis of proportion has been conducted [Murad MH, Sultan S, Haffar S, Bazerbachi F. Methodological quality and synthesis of case series and case reports. BMJ Evid Based Med. 2018 Apr;23(2):60-63. doi: 10.1136/bmjebm-2017-110853. Epub 2018 Feb 2. PMID: 29420178; PMCID: PMC6234235.]. To draw statistical inferences from heterogeneous studies, we employed non-iterative estimate of the inter-study variance component based on a random effects model (s2), taking into account that statistical heterogeneity is believed to be due to clinical diversity. Tau^2 = 0 indicates no between-study heterogeneity. Double arcsine transformations have been applied to the observed proportions identified across a collection of studies to make the transformed proportions follow a normal distribution in order to accurately estimate the summary proportion and increase the validity of the associated statistical analyses. Multiple meta-regression with the Knapp-Hartung adjustment has been used to test the influence of the publication date and Covid-19 on early mortality. A p value of 0.05 was considered statistically significant.
Statistical analyses have been done using the packages "meta" and "metafor" of R software, version 4.0.5.

PICOS criteria for inclusion and exclusion of studies into meta-analysis.

Parameter	Inclusion criteria	Exclusion criteria
**Patients**	Adult patients (≥18 years) with mechanical complications after acute myocardial infarction.	Patients without mechanical complications after acute myocardial infarction
**Intervention**	Surgery<br>Coservative treatment	-
**Comparator**	Surgery vs Coservative treatment<br>Covid-19 pandemic vs before	
**Outcomes**	Primary: early mortality.	-
**Study design**	Case reports<br>Case series<br>Controlled before-and-after studies<br>Prospective and retrospective cohort studies<br>Cross-sectional studies<br>Case-control studies	Repeat publications of the same analysis or dataset<br>Conference abstracts<br>Editorials & opinion pieces<br>Books or grey literature<br>Restrictions: English language

Quality assessment of the included studies.

Study (Author/Year)	Newcastle-Ottawa Scale[111]									Quality Rating Scheme for Studies and Other Evidence (1 best to 5 low)
	Representativeness	Selection	Ascertainment of exposure	Outcome of interest was not present at start of study	Comparability	Assessment of outcome	Follow-up long enough	Adeguacy of follow-up	Quality score	
Bouchart, 1998	✶			✶		✶	✶	✶	5	4
David, 1998	✶		✶	✶		✶	✶	✶	6	4
Menon, 2000	✶		✶	✶		✶	✶	✶	6	4
Crenshaw, 2000	✶		✶	✶		✶	✶	✶	6	4
Deja, 2000	✶		✶	✶		✶	✶		5	4
Chessa, 2002	✶		✶	✶		✶	✶		4	4
Labrousse, 2002	✶		✶	✶			✶	✶	5	4
Szkutnick, 2003				✶			✶		2	4
Goldstein, 2003	✶		✶	✶		✶	✶	✶	4	4
Barker, 2003	✶		✶	✶		✶	✶	✶	6	4
Cerin, 2003	✶		✶	✶		✶	✶	✶	6	4
Thiele, 2003	✶		✶	✶		✶	✶	✶	6	4
Holzer, 2004	✶		✶	✶		✶	✶	✶	6	4
Jeppsson, 2005	✶		✶	✶		✶	✶	✶	6	4
Demkow, 2005	✶		✶	✶		✶	✶	✶	6	4
Mantovani, 2006	✶		✶	✶		✶	✶	✶	6	4
Marianakis, 2007	✶		✶	✶		✶	✶		5	4
Martinez, 2007	✶		✶	✶		✶	✶	✶	6	4
Kaulfersch, 2007	✶		✶	✶		✶	✶	✶	6	4
Ahmed, 2008	✶		✶	✶		✶	✶	✶	6	4
Poulsen, 2008	✶		✶	✶		✶	✶	✶	6	4
Sajja, 2008	✶			✶			✶		3	4
Maltais, 2009	✶			✶			✶	✶	4	4
Thiele, 2009	✶		✶	✶		✶	✶	✶	6	4
Coskun, 2009	✶			✶		✶	✶	✶	5	4
Moreyra, 2010	✶		✶	✶		✶	✶	✶	6	4
Fukushima, 2010	✶		✶	✶		✶	✶	✶	6	4
Murashita, 2010	✶		✶	✶		✶	✶	✶	6	4
Sa, 2010	✶		✶	✶		✶	✶	✶	6	4
Sibal, 2010	✶		✶	✶		✶	✶	✶	6	4
Arnaoutakis, 2012	✶		✶	✶		✶	✶	✶	6	4
Abu-Omar, 2012	✶		✶	✶		✶	✶		5	4

Assenza, 2013	✶		✶	✶		✶	✶	✶	6	4
Zhu, 2013	✶		✶	✶		✶	✶	✶	6	4
Morillon-Luton, 2013	✶		✶	✶		✶	✶	✶	6	4
Hu, 2013	✶			✶		✶	✶	✶	5	4
Kettner, 2013	✶		✶	✶		✶	✶	✶	6	4
Pang, 2013	✶		✶	✶		✶	✶	✶	6	4
Park, 2013	✶			✶		✶	✶		4	4
Rohn, 2013	✶		✶	✶		✶	✶	✶	6	4
Yam, 2013	✶		✶	✶		✶	✶	✶	6	4
Xu, 2014	✶		✶	✶		✶	✶	✶	6	4
Heiberg, 2014	✶		✶	✶		✶	✶	✶	6	4
Lundblad, 2014	✶		✶	✶		✶	✶	✶	6	4
Egbe, 2015	✶		✶	✶		✶	✶		5	4
Tang, 2015	✶		✶	✶		✶	✶	✶	6	4
Trivedi, 2015	✶		✶	✶		✶	✶		5	4
Huang, 2015	✶		✶	✶		✶	✶	✶	6	4
Kim, 2015	✶		✶	✶		✶	✶		5	4
Takahashi, 2015	✶			✶		✶	✶		4	4
Sulzberg, 2016	✶		✶	✶		✶	✶	✶	6	4
Cinq-Mars, 2016	✶		✶	✶		✶	✶	✶	6	4
Yalcinkaya, 2016	✶		✶	✶		✶	✶		5	4
Nie, 2017	✶		✶	✶		✶	✶	✶	6	4
Sabiniewicz, 2017	✶		✶	✶		✶	✶	✶	6	4
Premchand, 2017	✶			✶		✶	✶		4	4
Malhotra, 2017	✶		✶	✶		✶	✶		5	4
Abbasnejad, 2018	✶		✶	✶		✶	✶		5	4
Khan, 2018	✶		✶	✶		✶	✶		5	4
Pojar, 2018	✶		✶	✶		✶	✶		5	4
Elbadawi, 2019	✶		✶	✶		✶	✶	✶	6	4
Dogra, 2019	✶		✶	✶		✶	✶		5	4
Li, 2019	✶			✶		✶	✶		5	4
Sakaguchi, 2019	✶		✶	✶		✶	✶	✶	6	4
Ariza-Solè, 2020	✶		✶	✶		✶	✶	✶	6	4
Sanmartín-Fernández, 2020	✶		✶	✶		✶	✶	✶	6	4
Pahuja, 2020	✶		✶	✶		✶	✶	✶	6	4
Fujita, 2020	✶		✶	✶		✶	✶	✶	6	4
Kilic, 2020	✶		✶	✶		✶	✶	✶	6	4
Bhardwaj, 2020	✶		✶	✶		✶	✶	✶	6	4

It is assigned a maximum score of one star for each category, except for comparability that can be awarded to a maximum of two stars.

Characteristics of the included studies during Covid-19 pandemic.

Study (Year of Publication, First Author, [Reference Number])	Country	Design and Study Period	Sample Size (n)	Age (mean±sd)	Male (n/%)	Type of mechanical complication	Surgery	Type of surgical treatment	Conservative treatment
2020, Pilato Emanuele, 32980627	Italy	Case series	5	54,8	nr	Free wall rupture	1/5	Jatene	4/5
2020, Atreya Auras R, 32839759	United States	Case report	1	52	1/100%	Papillary muscle rupture	Yes	Mitral Valve Replacement	no
2021, Briosa e Gala Andre, 33153833	United Kingdom	Case report	1	69	0/0%	Papillary muscle rupture	No		yes
2020, Tan Joo Hor, 33733041	Singapore	Case report	1	65	1/100%	Free wall rupture	Yes	Patch repair	no
2021, Evans Matthew C, 33733042	United States	Case report	1	37	1/100%	Septal rupture	No		yes
2020, Masroor Saqib, 32419177	United States	Case report	1	48	1/100%	Septal rupture	Yes	Septal rupture repair	no
2020, Albiero Remo, 32835259	Italy	Case report	1	72	1/100%	Free wall rupture	Yes	Patch repair	no
2020, Rimac Goran, 32498079	Canada	Case report	1	64	0/0%	LV pseudoaneurysm	Yes	Patch repair	no
2020, Kassimis George, 32819494	Greece	Case report	1	71	0/0%	Free wall rupture	No		yes
2020, Shah Kulin, 32839760	United States	Case series	1	75	0/0%	Septal rupture	Yes	Patch repair	no
2020, Elkattawy Sherif, 32944444	United States	Case report	1	65	1/100%	Septal rupture	No		yes
2021, Kitahara Satoshi, 33547221	Japan	Retrospective study	9	75,4	3/33,3%		Yes		No
2021, Allende Norberto Gustavo, 33599346	Argentina	Case report	2	65,5	1/50%	LV pseudoaneurysm	No		Yes
2021, Bakhshi Hooman, 33387761	United States	Case report	2	54,5	2/100%	Septal rupture	Yes	Heart Trasplantation	No
2020, Alsidawi Said, 32835258	United States	Case report	2	64,5	0/0%	Septal rupture	1/2	Patch repair	1/2
2020, Parikh Malav, 32989437	United States	Case report	2	63,5	1/50%	Septal rupture	1/2	Patch repair	1/2
2021, Lin Ting-Wei, 33488037	Taiwan	Cases series	5	69,6	3/60%		5/5		5/5
2020, Yousif Nooraldeam, 33154939	Kingdom of Bahrain	Case report	1	53	1/100%	Papillary muscle rupture	Yes	Mitral valve replacement	No
2020, Joshi Saurabh, 32835262	United States	Case report	1	72	0/0%	Septal rupture	No		Yes
2021, Chhetry Minar, 34383957	United States	Case report	1	71	1/100%	Septal rupture	Yes	Septal rupture repair	No
2021, Gadre Akshaya, 34221777	United States	Case report	1	66	0/0%	Septal rupture	No		Yes
2020, Ahmed Taha, 32839758	United States	Case report	1	65	1/100%	Septal rupture	Yes	Septal rupture repair	No
2021, Goraya Muhammad Hassan Naeem, 34259086	Pakistan	Case report	1	53	1/100%	Septal rupture	Yes	Septal rupture repair	No
2021, Qureshi Waqas T, 32863191	United States	Case series	4	66,25	1/25%		4/4		No
2020, Gaballa Salem, 32724754	United States	Case report	1	87	0/100%	Septal rupture	No		Yes
2020, Kunkel Katherine J, 33521677	United States	Case report	1	57	1/100%	Papillary muscle rupture	Yes	Mitral valve replacement	No
2021, Nasr George H, 33888133	United States	Case report	1	67	1/100%	Free wall rupture			
2020, Khanal Suraj, 33274165	India	Case report	1	61	1/100%	Septal rupture	Yes	Patch repair	No
2021, Briani Martina, 34104861	Italy	Case report	1	70	1/100%	LV aneurysm	Yes	Patch repair	No
2021, Fardman Alexandre, 34143840	Israel	Prospective multicenter study	14	nr	nr	Mechanical complication	1/14	nr	13/14

Supplementary References.

Bouchart F, Bessou JP, Tabley A, Redonnet M, Mouton-Schleifer D, Haas-Hubscher C, Soyer R. Urgent surgical repair of postinfarction ventricular septal rupture: early and late outcome. J Card Surg. 1998 Mar;13(2):104-12. doi: 10.1111/j.1540-8191.1998.tb01242.x. PMID: 10063955.

David TE, Armstrong S. Surgical repair of postinfarction ventricular septal defect by infarct exclusion. Semin Thorac Cardiovasc Surg. 1998 Apr;10(2):105-10. doi: 10.1016/s1043-0679(98)70003-6. PMID: 9620457.

Menon V, Webb JG, Hillis LD, Sleeper LA, Abboud R, Dzavik V, Slater JN, Forman R, Monrad ES, Talley JD, Hochman JS. Outcome and profile of ventricular septal rupture with cardiogenic shock after myocardial infarction: a report from the SHOCK Trial Registry. SHould we emergently revascularize Occluded Coronaries in cardiogenic shocK? J Am Coll Cardiol. 2000 Sep;36(3 Suppl A):1110-6. doi: 10.1016/s0735-1097(00)00878-0. PMID: 10985713.

Crenshaw BS, Granger CB, Birnbaum Y, et al. Risk factors, angiographic patterns, and outcomes in patients with ventricular septal defect complicating acute myocardial infarction. GUSTO-I (Global Utilization of Streptokinase and TPA for Occluded Coronary Arteries) Trial Investigators. Circulation. 2000 Jan 4-11;101(1):27-32

Birnbaum Y, Wagner GS, Gates KB, Thompson TD, Barbash GI, Siegel RJ, Granger CB, Fishbein MC, Crenshaw BS, Califf RM. Clinical and electrocardiographic variables associated with increased risk of ventricular septal defect in acute anterior myocardial infarction. Am J Cardiol. 2000 Oct 15;86(8):830-4. doi: 10.1016/s0002-9149(00)01101-2. PMID: 11024396.

Deja MA, Szostek J, Widenka K, Szafron B, Spyt TJ, Hickey MS, Sosnowski AW. Post infarction ventricular septal defect - can we do better? Eur J Cardiothorac Surg. 2000 Aug;18(2):194-201. doi: 10.1016/s1010-7940(00)00482-6. PMID: 10925229.

Chessa M, Carminati M, Cao QL, Butera G, Giusti S, Bini RM, Hijazi ZM. Transcatheter closure of congenital and acquired muscular ventricular septal defects using the Amplatzer device. J Invasive Cardiol. 2002 Jun;14(6):322-7. PMID: 12042624.

Labrousse L, Choukroun E, Chevalier JM, Madonna F, Robertie F, Merlico F, Coste P, Deville C. Surgery for post infarction ventricular septal defect (VSD): risk factors for hospital death and long term results. Eur J Cardiothorac Surg. 2002 Apr;21(4):725-31; discussion 731-2. doi: 10.1016/s1010-7940(02)00054-4. PMID: 11932175.

Szkutnik M, Bialkowski J, Kusa J, Banaszak P, Baranowski J, Gasior M, Chodor P, Zembala M. Postinfarction ventricular septal defect closure with Amplatzer occluders. Eur J Cardiothorac Surg. 2003 Mar;23(3):323-7. doi: 10.1016/s1010-7940(02)00812-6. PMID: 12614801.

Goldstein JA, Casserly IP, Balzer DT, Lee R, Lasala JM. Transcatheter closure of recurrent postmyocardial infarction ventricular septal defects utilizing the Amplatzer postinfarction VSD device: a case series. Catheter Cardiovasc Interv. 2003 Jun;59(2):238-43. doi: 10.1002/ccd.10510. PMID: 12772250.

Barker TA, Ramnarine IR, Woo EB, Grayson AD, Au J, Fabri BM, Bridgewater B, Grotte GJ. Repair of post-infarct ventricular septal defect with or without coronary artery bypass grafting in the northwest of England: a 5-year multi-institutional experience. Eur J Cardiothorac Surg. 2003 Dec;24(6):940-6. doi: 10.1016/s1010-7940(03)00465-2. PMID: 14643812.

Cerin G, Di Donato M, Dimulescu D, Montericcio V, Menicanti L, Frigiola A, De Ambroggi L. Surgical treatment of ventricular septal defect complicating acute myocardial infarction. Experience of a north Italian referral hospital. Cardiovasc Surg. 2003 Apr;11(2):149-54. doi: 10.1016/s0967-2109(02)00190-4. PMID: 12664051.

Thiele H, Lauer B, Hambrecht R, Boudriot E, Sick P, Niebauer J, Falk V, Schuler G. Short- and long-term hemodynamic effects of intra-aortic balloon support in ventricular septal defect complicating acute myocardial infarction. Am J Cardiol. 2003 Aug 15;92(4):450-4. doi: 10.1016/s0002-9149(03)00665-9. PMID: 12918526.

Holzer R, Balzer D, Amin Z, Ruiz CE, Feinstein J, Bass J, Vance M, Cao QL, Hijazi ZM. Transcatheter closure of postinfarction ventricular septal defects using the new Amplatzer muscular VSD occluder: Results of a U.S. Registry. Catheter Cardiovasc Interv. 2004 Feb;61(2):196-201. doi: 10.1002/ccd.10784. PMID: 14755811.

Jeppsson A, Liden H, Johnsson P, Hartford M, Rådegran K. Surgical repair of post infarction ventricular septal defects: a national experience. Eur J Cardiothorac Surg. 2005 Feb;27(2):216-21. doi: 10.1016/j.ejcts.2004.10.037. PMID: 15691673.

Demkow M, Ruzyllo W, Kepka C, Chmielak Z, Konka M, Dzielinska Z, Wilczynski J, Juraszynski Z. Primary transcatheter closure of postinfarction ventricular septal defects with the Amplatzer septal occluder- immediate results and up-to 5 years follow-up. EuroIntervention. 2005 May;1(1):43-7. PMID: 19758875.

Mantovani V, Mariscalco G, Leva C, Blanzola C, Sala A. Surgical repair of post-infarction ventricular septal defect: 19 years of experience. Int J Cardiol. 2006 Apr 4;108(2):202-6. doi: 10.1016/j.ijcard.2005.05.007. Epub 2005 Jun 13. PMID: 15950300.

Marinakis A, Vydt T, Dens J, Gewillig M, Van Deyk K, Budts W. Percutaneous transcatheter ventricular septal defect closure in adults with Amplatzer septal occluders. Acta Cardiol. 2007 Aug;62(4):391-5. doi: 10.2143/AC.62.4.2022283. PMID: 17824300.

Martinez MW, Mookadam F, Sun Y, Hagler DJ. Transcatheter closure of ischemic and post-traumatic ventricular septal ruptures. Catheter Cardiovasc Interv. 2007 Feb 15;69(3):403-7. doi: 10.1002/ccd.20949. PMID: 17195200.

Kaulfersch C, Daehnert I, Schuler G, Thiele H. Transcatheter closure of postinfarction ventricular septal defects. Minerva Cardioangiol. 2007 Oct;55(5):693-701. PMID: 17912172.

Ahmed J, Ruygrok PN, Wilson NJ, Webster MW, Greaves S, Gerber I. Percutaneous closure of post-myocardial infarction ventricular septal defects: a single centre experience. Heart Lung Circ. 2008 Apr;17(2):119-23. doi: 10.1016/j.hlc.2007.09.001. Epub 2007 Dec 3. PMID: 18060839.

Poulsen SH, Praestholm M, Munk K, Wierup P, Egeblad H, Nielsen-Kudsk JE. Ventricular septal rupture complicating acute myocardial infarction: clinical characteristics and contemporary outcome. Ann Thorac Surg. 2008 May;85(5):1591-6. doi: 10.1016/j.athoracsur.2008.01.010. PMID: 18442545.

Sajja LR, Mannam GC, Gutti RS, Goli NR, Sompalli S, Penumatsa RR. Postinfarction ventricular septal defect: patch repair with infarct exclusion. Asian Cardiovasc Thorac Ann. 2008 Jun;16(3):215-20. doi: 10.1177/021849230801600308. PMID: 18515671.

Maltais S, Ibrahim R, Basmadjian AJ, Carrier M, Bouchard D, Cartier R, Demers P, Ladouceur M, Pellerin M, Perrault LP. Postinfarction ventricular septal defects: towards a new treatment algorithm? Ann Thorac Surg. 2009 Mar;87(3):687-92. doi: 10.1016/j.athoracsur.2008.11.052. PMID: 19231370.

Thiele H, Kaulfersch C, Daehnert I, Schoenauer M, Eitel I, Borger M, Schuler G. Immediate primary transcatheter closure of postinfarction ventricular septal defects. Eur Heart J. 2009 Jan;30(1):81-8. doi: 10.1093/eurheartj/ehn524. Epub 2008 Nov 25. PMID: 19036747.

Coskun KO, Coskun ST, Popov AF, Hinz J, Schmitto JD, Bockhorst K, Stich KM, Koerfer R. Experiences with surgical treatment of ventricle septal defect as a post infarction complication. J Cardiothorac Surg. 2009 Jan 6;4:3. doi: 10.1186/1749-8090-4-3. PMID: 19126196; PMCID: PMC2631454.

Moreyra AE, Huang MS, Wilson AC, Deng Y, Cosgrove NM, Kostis JB; MIDAS Study Group (MIDAS 13). Trends in incidence and mortality rates of ventricular septal rupture during acute myocardial infarction. Am J Cardiol. 2010 Oct 15;106(8):1095-100. doi: 10.1016/j.amjcard.2010.06.013. PMID: 20920645.

Fukushima S, Tesar PJ, Jalali H, Clarke AJ, Sharma H, Choudhary J, Bartlett H, Pohlner PG. Determinants of in-hospital and long-term surgical outcomes after repair of postinfarction ventricular septal rupture. J Thorac Cardiovasc Surg. 2010 Jul;140(1):59-65. doi: 10.1016/j.jtcvs.2009.09.018. Epub 2009 Nov 17. PMID: 19919868.

Murashita T, Komiya T, Tamura N, Sakaguchi G, Kobayashi T, Sunagawa G. The clinical challenge to reduce the postoperative residual shunt in surgical repair of postinfarction ventricular septal perforation. Interact Cardiovasc Thorac Surg. 2010 Jul;11(1):38-41. doi: 10.1510/icvts.2009.229179. Epub 2010 Apr 9. PMID: 20382679.

Sá MP, Sá MV, Barbosa CH, Silva NP, Escobar RR, Rueda FG, Silva FP, Lima Rde C. Clinical and surgical profile of patients operated for postinfarction interventricular septal rupture. Rev Bras Cir Cardiovasc. 2010 Jul-Sep;25(3):341-9. English, Portuguese. doi: 10.1590/s0102-76382010000300010. PMID: 21103742.

Sibal AK, Prasad S, Alison P, Nand P, Haydock D. Acute ischaemic ventricular septal defect--a formidable surgical challenge. Heart Lung Circ. 2010 Feb;19(2):71-4. doi: 10.1016/j.hlc.2009.09.004. Epub 2009 Nov 14. PMID: 19914868.

Arnaoutakis GJ, Zhao Y, George TJ, Sciortino CM, McCarthy PM, Conte JV. Surgical repair of ventricular septal defect after myocardial infarction: outcomes from the Society of Thoracic Surgeons National Database. Ann Thorac Surg. 2012 Aug;94(2):436-43; discussion 443-4. doi: 10.1016/j.athoracsur.2012.04.020. Epub 2012 May 23. PMID: 22626761; PMCID: PMC3608099.

Abu-Omar Y, Bhinda P, Choong CK, Nashef SA, Nair S. Survival after surgical repair of ischemic ventricular septal rupture. Asian Cardiovasc Thorac Ann. 2012 Aug;20(4):404-8. doi: 10.1177/0218492312438739. PMID: 22879546.

Assenza GE, McElhinney DB, Valente AM, Pearson DD, Volpe M, Martucci G, Landzberg MJ, Lock JE. Transcatheter closure of post-myocardial infarction ventricular septal rupture. Circ Cardiovasc Interv. 2013 Feb;6(1):59-67. doi: 10.1161/CIRCINTERVENTIONS.112.972711. Epub 2013 Jan 22. PMID: 23339839.

Zhu XY, Qin YW, Han YL, Zhang DZ, Wang P, Liu YF, Xu YW, Jing QM, Xu K, Gersh BJ, Wang XZ. Long-term efficacy of transcatheter closure of ventricular septal defect in combination with percutaneous coronary intervention in patients with ventricular septal defect complicating acute myocardial infarction: a multicentre study. EuroIntervention. 2013 Mar;8(11):1270-6. doi: 10.4244/EIJV8I11A195. PMID: 23538156.

Morillon-Lutun S, Maucort-Boulch D, Mewton N, Farhat F, Bresson D, Girerd N, Desebbe O, Henaine R, Kirkorian G, Bonnefoy-Cudraz E. Therapeutic management changes and mortality rates over 30 years in ventricular septal rupture complicating acute myocardial infarction. Am J Cardiol. 2013 Nov 1;112(9):1273-8. doi: 10.1016/j.amjcard.2013.06.009. Epub 2013 Jul 16. PMID: 23866732.

Hu XY, Qiu H, Qiao SB, Kang LM, Song L, Zhang J, Tan XY, Wu Y, Yang YJ, Gao RL, Chen ZJ. Clinical analysis and risk stratification of ventricular septal rupture following acute myocardial infarction. Chin Med J (Engl). 2013 Nov;126(21):4105-8. PMID: 24229682.

Kettner J, Sramko M, Holek M, Pirk J, Kautzner J. Utility of intra-aortic balloon pump support for ventricular septal rupture and acute mitral regurgitation complicating acute myocardial infarction. Am J Cardiol. 2013 Dec 1;112(11):1709-13. doi: 10.1016/j.amjcard.2013.07.035. Epub 2013 Sep 13. PMID: 24035169.

Pang PY, Sin YK, Lim CH, Tan TE, Lim SL, Chao VT, Su JW, Chua YL. Outcome and survival analysis of surgical repair of post-infarction ventricular septal rupture. J Cardiothorac Surg. 2013 Mar 9;8:44. doi: 10.1186/1749-8090-8-44. PMID: 23497648; PMCID: PMC3599964.

Park SJ, Kim JB, Jung SH, Choo SJ, Chung CH, Lee JW. Surgical Repair of Ventricular Septal Defect after Myocardial Infarction: A Single Center Experience during 22 Years. Korean J Thorac Cardiovasc Surg. 2013 Dec;46(6):433-8. doi: 10.5090/kjtcs.2013.46.6.433. Epub 2013 Dec 6. PMID: 24368969; PMCID: PMC3868690.

Rohn V, Grus T, Lindner J, Lipš M, Bělohlávek J. Postinfarction ventricular septal rupture - a rare complication remains challenge for cardiac surgical team. Prague Med Rep. 2013;114(1):9-17. doi: 10.14712/23362936.2014.33. PMID: 23547721.

Yam N, Au TW, Cheng LC. Post-infarction ventricular septal defect: surgical outcomes in the last decade. Asian Cardiovasc Thorac Ann. 2013 Oct;21(5):539-45. doi: 10.1177/0218492312462041. Epub 2013 Jul 9. PMID: 24570555.

Heiberg J, Hjortdal VE, Nielsen-Kudsk JE. Long-term outcome after transcatheter closure of postinfarction ventricular septal rupture. J Interv Cardiol. 2014 Oct;27(5):509-15. doi: 10.1111/joic.12146. Epub 2014 Aug 25. PMID: 25155883.

Lundblad R, Abdelnoor M. Surgery of postinfarction ventricular septal rupture: the effect of David infarct exclusion versus Daggett direct septal closure on early and late outcomes. J Thorac Cardiovasc Surg. 2014 Dec;148(6):2736-42. doi: 10.1016/j.jtcvs.2014.06.076. Epub 2014 Jul 22. PMID: 25135234.

Egbe AC, Poterucha JT, Rihal CS, Taggart NW, Cetta F, Cabalka AK, Pollak PM, Reeder GS, Hagler DJ. Transcatheter closure of postmyocardial infarction, iatrogenic, and postoperative ventricular septal defects: The Mayo Clinic experience. Catheter Cardiovasc Interv. 2015 Dec 1;86(7):1264-70. doi: 10.1002/ccd.25989. Epub 2015 May 29. PMID: 26033272.

Tang L, Fang Z, Hu X, Tang J, Shen X, Lu X, Zhao Y, Li J, Zhou S. Non-surgical repair of ventricular septal rupture after acute myocardial infarction. Int J Cardiol. 2015 Apr 15;185:328-32. doi: 10.1016/j.ijcard.2015.03.144. Epub 2015 Mar 17. PMID: 25828675.

Trivedi KR, Aldebert P, Riberi A, Mancini J, Levy G, Macia JC, Quilicci J, Habib G, Fraisse A. Sequential management of post-myocardial infarction ventricular septal defects. Arch Cardiovasc Dis. 2015 May;108(5):321-30. doi: 10.1016/j.acvd.2015.01.005. Epub 2015 Mar 6. PMID: 25754906.

Huang SM, Huang SC, Wang CH, Wu IH, Chi NH, Yu HY, Hsu RB, Chang CI, Wang SS, Chen YS. Risk factors and outcome analysis after surgical management of ventricular septal rupture complicating acute myocardial infarction: a retrospective analysis. J Cardiothorac Surg. 2015 May 4;10:66. doi: 10.1186/s13019-015-0265-2. PMID: 25935413; PMCID: PMC4426168.

Kim IS, Lee JH, Lee DS, Cho YH, Kim WS, Jeong DS, Lee YT. Surgical Outcomes of a Modified Infarct Exclusion Technique for Post-Infarction Ventricular Septal Defects. Korean J Thorac Cardiovasc Surg. 2015 Dec;48(6):381-6. doi: 10.5090/kjtcs.2015.48.6.381. Epub 2015 Dec 5. PMID: 26665103; PMCID: PMC4672971.

Takahashi H, Arif R, Almashhoor A, Ruhparwar A, Karck M, Kallenbach K. Long-term results after surgical treatment of postinfarction ventricular septal rupture. Eur J Cardiothorac Surg. 2015 Apr;47(4):720-4. doi: 10.1093/ejcts/ezu248. Epub 2014 Jul 3. PMID: 24994755.

Cinq-Mars A, Voisine P, Dagenais F, Charbonneau É, Jacques F, Kalavrouziotis D, Perron J, Mohammadi S, Dubois M, Le Ven F, Poirier P, O'Connor K, Bernier M, Bergeron S, Sénéchal M. Risk factors of mortality after surgical correction of ventricular septal defect following myocardial infarction: Retrospective analysis and review of the literature. Int J Cardiol. 2016 Mar 1;206:27-36. doi: 10.1016/j.ijcard.2015.12.011. Epub 2015 Dec 14. PMID: 26773765.

Yalçınkaya A, Lafçı G, Diken Aİ, Aksoy E, Çiçek ÖF, Lafçı A, Korkmaz K, Çağlı K. Early Mortality and Long-term Survival after Repair of Post-infarction Ventricular Septal Rupture: An Institutional Report of Experience. Heart Lung Circ. 2016 Apr;25(4):384-91. doi: 10.1016/j.hlc.2015.08.016. Epub 2015 Oct 9. PMID: 26530437.

Nie YL, Lin MC, Lin WW, Wang CC, Chen CP, Lin CH, Shyu TC, Quek YW, Jan SL, Fu YC. Transcatheter device closure of postmyocardial infarction ventricular septal defect. J Chin Med Assoc. 2017 Jan;80(1):34-38. doi: 10.1016/j.jcma.2016.02.014. Epub 2016 Nov 23. PMID: 27889458.

Sabiniewicz R, Huczek Z, Zbroński K, Scisło P, Rymuza B, Kochman J, Marć M, Grygier M, Araszkiewicz A, Dziarmaga M, Leśniewicz P, Hiczkiewicz J, Kidawa M, Filipiak KJ, Opolski G. Percutaneous Closure of Post-Infarction Ventricular Septal Defects-An Over Decade-long Experience. J Interv Cardiol. 2017 Feb;30(1):63-71. doi: 10.1111/joic.12367. Epub 2017 Jan 11. PMID: 28078714.

Premchand RK, Garipalli R, Padmanabhan TN, Manik G. Percutaneous closure of post-myocardial infarction ventricular septal rupture - A single centre experience. Indian Heart J. 2017 Apr;69 Suppl 1(Suppl 1):S24-S27. doi: 10.1016/j.ihj.2016.10.004. Epub 2016 Nov 1. PMID: 28400035; PMCID: PMC5388014.

Malhotra A, Patel K, Sharma P, Wadhawa V, Madan T, Khandeparkar J, Shah K, Patel S. Techniques, Timing & Prognosis of Post Infarct Ventricular Septal Repair: a Re-look at Old Dogmas. Braz J Cardiovasc Surg. 2017 May-Jun;32(3):147-155. doi: 10.21470/1678-9741-2016-0032. PMID: 28832791; PMCID: PMC5570397.

Abbasnejad M, Vand MT, Khamnian Z, Separham A. In-hospital outcome of patients with post-MI VSD: a single-center study. Kardiochir Torakochirurgia Pol. 2018 Dec;15(4):227-232. doi: 10.5114/kitp.2018.80918. Epub 2018 Dec 31. PMID: 30647745; PMCID: PMC6329881.

Khan MY, Waqar T, Qaisrani PG, Khan AZ, Khan MS, Zaman H, Jalal A. Surgical Repair of post-infarction ventricular septal rupture: Determinants of operative mortality and survival outcome analysis. Pak J Med Sci. 2018 Jan-Feb;34(1):20-26. doi: 10.12669/pjms.341.13906. PMID: 29643872; PMCID: PMC5857013.

Pojar M, Harrer J, Omran N, Turek Z, Striteska J, Vojacek J. Surgical treatment of postinfarction ventricular septal defect: risk factors and outcome analysis. Interact Cardiovasc Thorac Surg. 2018 Jan 1;26(1):41-46. doi: 10.1093/icvts/ivx230. PMID: 29049690.

Elbadawi A, Elgendy IY, Mahmoud K, Barakat AF, Mentias A, Mohamed AH, Ogunbayo GO, Megaly M, Saad M, Omer MA, Paniagua D, Abbott JD, Jneid H. Temporal Trends and Outcomes of Mechanical Complications in Patients With Acute Myocardial Infarction. JACC Cardiovasc Interv. 2019 Sep 23;12(18):1825-1836. doi: 10.1016/j.jcin.2019.04.039. PMID: 31537282.

Dogra N, Puri GD, Thingnam SKS, Arya VK, Kumar B, Mahajan S, Verma M. Early thrombolysis is associated with decreased operative mortality in postinfarction ventricular septal rupture. Indian Heart J. 2019 May-Jun;71(3):224-228. doi: 10.1016/j.ihj.2019.04.011. Epub 2019 May 3. PMID: 31543194; PMCID: PMC6796617.

Li H, Zhang S, Yu M, Xu J, Dong C, Yang Y, Sun H, Song Y. Profile and Outcomes of Surgical Treatment for Ventricular Septal Rupture in Patients With Shock. Ann Thorac Surg. 2019 Oct;108(4):1127-1132. doi: 10.1016/j.athoracsur.2019.03.101. Epub 2019 May 7. PMID: 31075249.

Sakaguchi G, Miyata H, Motomura N, Ueki C, Fukuchi E, Yamamoto H, Takamoto S, Marui A. Surgical Repair of Post-Infarction Ventricular Septal Defect - Findings From a Japanese National Database. Circ J. 2019 Oct 25;83(11):2229-2235. doi: 10.1253/circj.CJ-19-0593. Epub 2019 Sep 11. PMID: 31511450.

Ariza-Solé A, Sánchez-Salado JC, Sbraga F, Ortiz D, González-Costello J, Blasco-Lucas A, Alegre O, Toral D, Lorente V, Santafosta E, Toscano J, Izquierdo A, Miralles A, Cequier Á. The role of perioperative cardiorespiratory support in post infarction ventricular septal rupture-related cardiogenic shock. Eur Heart J Acute Cardiovasc Care. 2020 Mar;9(2):128-137. doi: 10.1177/2048872618817485. Epub 2018 Dec 10. PMID: 30525871.

Sanmartín-Fernández M, Raposeiras-Roubin S, Anguita-Sánchez M, Marín F, Garcia-Marquez M, Fernández-Pérez C, Bernal-Sobrino JL, Elola-Somoza FJ, Bueno H, Cequier Á. In-hospital outcomes of mechanical complications in acute myocardial infarction: Analysis from a nationwide Spanish database. Cardiol J. 2021;28(4):589-597. doi: 10.5603/CJ.a2020.0181. Epub 2020 Dec 21. PMID: 33346367.

Pahuja M, Ranka S, Chauhan K, Patel A, Chehab O, Elmoghrabi A, Mony S, Ando T, Mishra T, Singh M, Abubaker H, Yassin A, Glazier JJ, Afonso L, Kapur NK, Burkhoff D. Rupture of Papillary Muscle and Chordae Tendinae Complicating STEMI: A Call for Action. ASAIO J. 2020 Oct 21. doi: 10.1097/MAT.0000000000001299. Epub ahead of print. PMID: 33093383.

Fujita T, Yamamoto H, Kobayashi J, Fukushima S, Miyata H, Yamashita K, Motomura N. Mitral valve surgery for ischemic papillary muscle rupture: outcomes from the Japan cardiovascular surgery database. Gen Thorac Cardiovasc Surg. 2020 Dec;68(12):1439-1446. doi: 10.1007/s11748-020-01418-y. Epub 2020 Jun 25. PMID: 32588291; PMCID: PMC7680308.

Kilic A, Sultan I, Chu D, Wang Y, Gleason TG. Mitral Valve Surgery for Papillary Muscle Rupture: Outcomes in 1342 Patients From The Society of Thoracic Surgeons Database. Ann Thorac Surg. 2020 Dec;110(6):1975-1981. doi: 10.1016/j.athoracsur.2020.03.097. Epub 2020 May 4. PMID: 32376352.

Bhardwaj B, Sidhu G, Balla S, Kumar V, Kumar A, Aggarwal K, Dohrmann ML, Alpert MA. Outcomes and Hospital Utilization in Patients With Papillary Muscle Rupture Associated With Acute Myocardial Infarction. Am J Cardiol. 2020 Apr 1;125(7):1020-1025. doi: 10.1016/j.amjcard.2019.12.051. Epub 2020 Jan 7. PMID: 31973809.

Sulzgruber P, El-Hamid F, Koller L, Forster S, Goliasch G, Wojta J, Niessner A. Long-term outcome and risk prediction in patients suffering acute myocardial infarction complicated by post-infarction cardiac rupture. Int J Cardiol. 2017 Jan 15;227:399-403. doi: 10.1016/j.ijcard.2016.11.037. Epub 2016 Nov 7. PMID: 27847155.

Figures

PRISMA flow chart of search strategy.

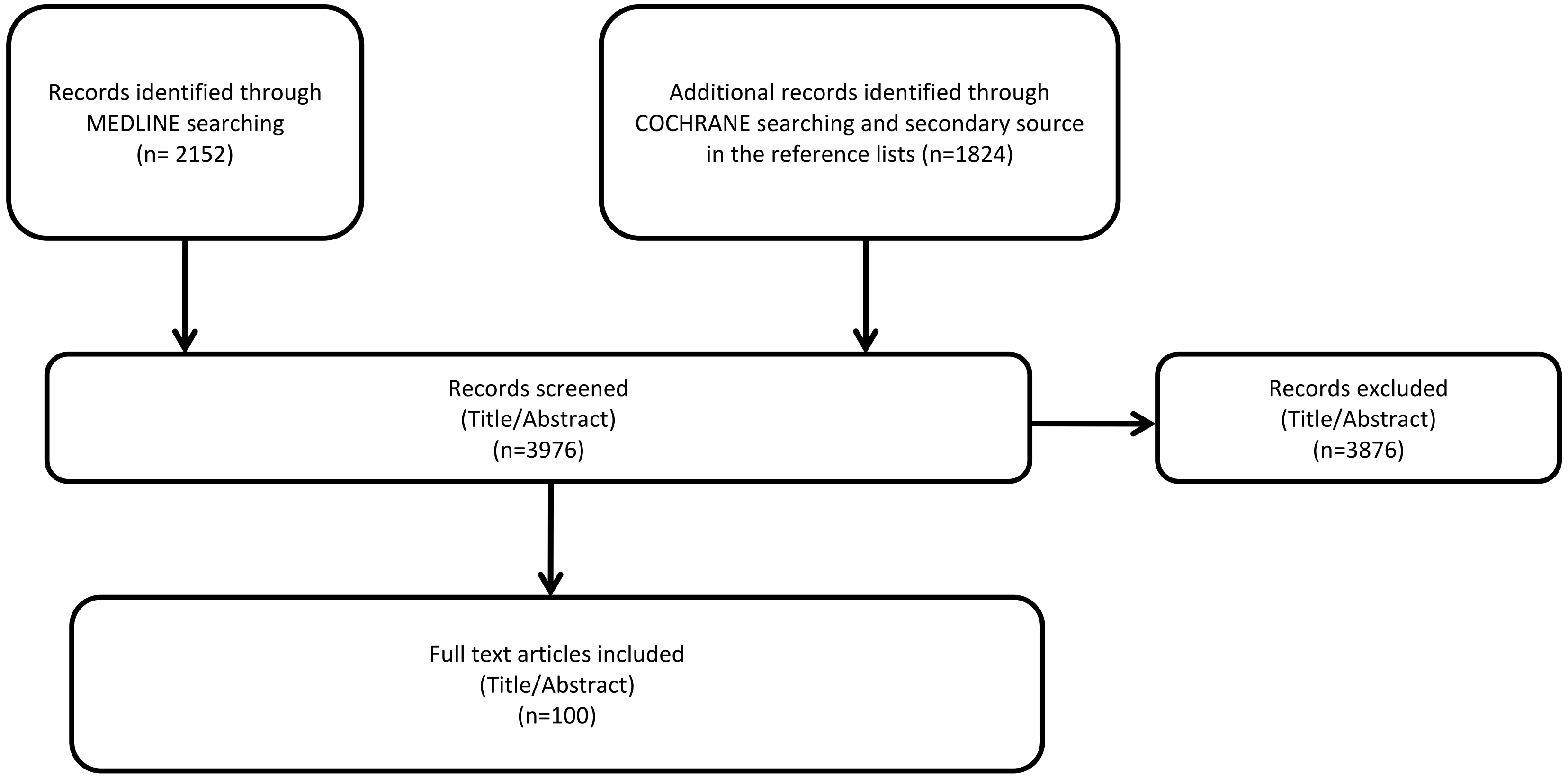

Pooled prevalence of early mortality during Covid-19 pandemic

Study	Cases	Total	Proportion	95% C.I.
Surgery				
Ahmed 2020	0	1	0.00	[0.00; 0.79]
Albiero 2020	1	1	1.00	[0.21; 1.00]
Alsidawi 2020	0	1	0.00	[0.00; 0.79]
Atreya 2020	1	1	1.00	[0.21; 1.00]
Bakhshi 2021	0	2	0.00	[0.00; 0.66]
Briani 2021	0	1	0.00	[0.00; 0.79]
Evans 2021	0	1	0.00	[0.00; 0.79]
Fardman 2021	2	2	1.00	[0.34; 1.00]
Goraya 2021	0	1	0.00	[0.00; 0.79]
Khanal 2020	0	1	0.00	[0.00; 0.79]
Kitahara 2021	0	7	0.00	[0.00; 0.35]
Kunkel 2020	0	1	0.00	[0.00; 0.79]
Lin 2020	3	5	0.60	[0.23; 0.88]
Masroor 2020	0	1	0.00	[0.00; 0.79]
Minar 2021	0	1	0.00	[0.00; 0.79]
Parikh 2020	0	1	0.00	[0.00; 0.79]
Pilato 2020	0	2	0.00	[0.00; 0.66]
Qureshi 2021	3	4	0.75	[0.30; 0.95]
Rimac 2020	0	1	0.00	[0.00; 0.79]
Shah 2020	0	1	0.00	[0.00; 0.79]
Tan 2020	0	1	0.00	[0.00; 0.79]
Yousif 2020	0	1	0.00	[0.00; 0.79]
**Fixed effect model**		.	**0.15**	**[0.01; 0.35]**
**Random effects**			**0.15**	**[0.01; 0.37]**
No surgery				
Allende 2021	0	2	0.00	[0.00; 0.66]
Alsidawi 2020	0	1	0.00	[0.00; 0.79]
Briosa e Gala 2020	1	1	1.00	[0.21; 1.00]
Elkattawy 2020	1	1	1.00	[0.21; 1.00]
Fardman 2021	4	13	0.31	[0.13; 0.58]
Gaballa 2020	1	1	1.00	[0.21; 1.00]
Gadre 2021	1	1	1.00	[0.21; 1.00]
Joshi 2020	1	1	1.00	[0.21; 1.00]
Kassimis 2020	1	1	1.00	[0.21; 1.00]
Kitahara 2021	2	2	1.00	[0.34; 1.00]
Nasr 2021	1	1	1.00	[0.21; 1.00]
Parikh 2020	1	1	1.00	[0.21; 1.00]
Pilato 2021	0	3	0.00	[0.00; 0.56]
**Fixed effect model**		.	**0.49**	**[0.24; 0.75]**
**Random effects**			**0.62**	**[0.27; 0.93]**
**Fixed effect model**		.	**0.28**	**[0.13; 0.45]**
**Random effects**			**0.31**	**[0.13; 0.52]**

Heterogeneity (Surgery): $I^2 = 9\%$, $\tau^2 = 0.0110$, $\chi^2_{21} = 23.01$ ($p = 0.34$)

Heterogeneity (No surgery): $I^2 = 35\%$, $\tau^2 = 0.0562$, $\chi^2_{12} = 18.54$ ($p = 0.10$)

Heterogeneity (overall): $I^2 = 26\%$, $\tau^2 = 0.0369$, $\chi^2_{34} = 45.79$ ($p = 0.09$)

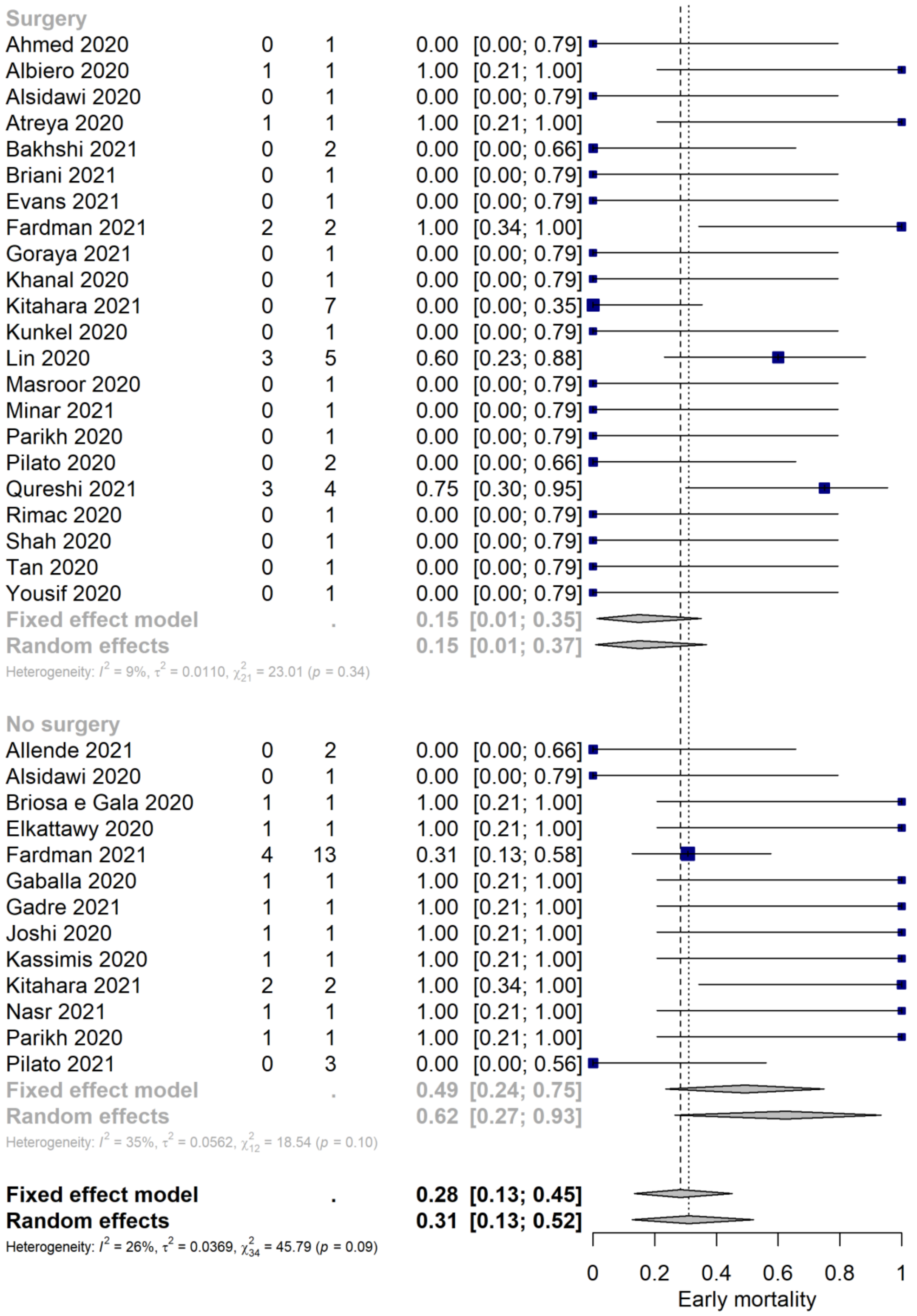

Funnel plot (A) and radial plot (B) for early mortality during Covid-19 pandemic showing no evidence of publication bias and heterogeneity among studies (Egger's test, P= 0.38), respectively.

A

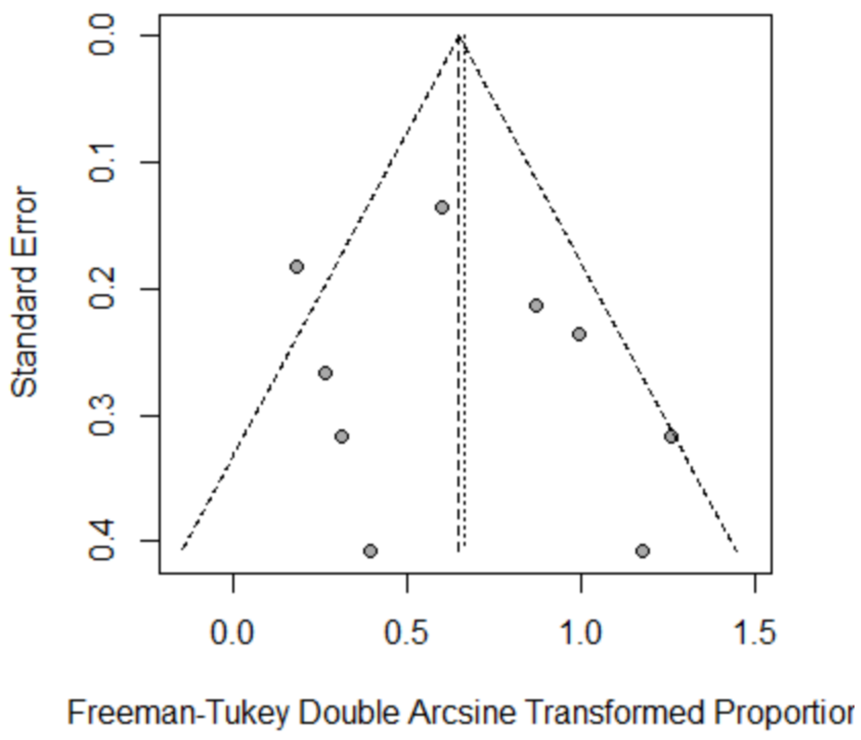

B

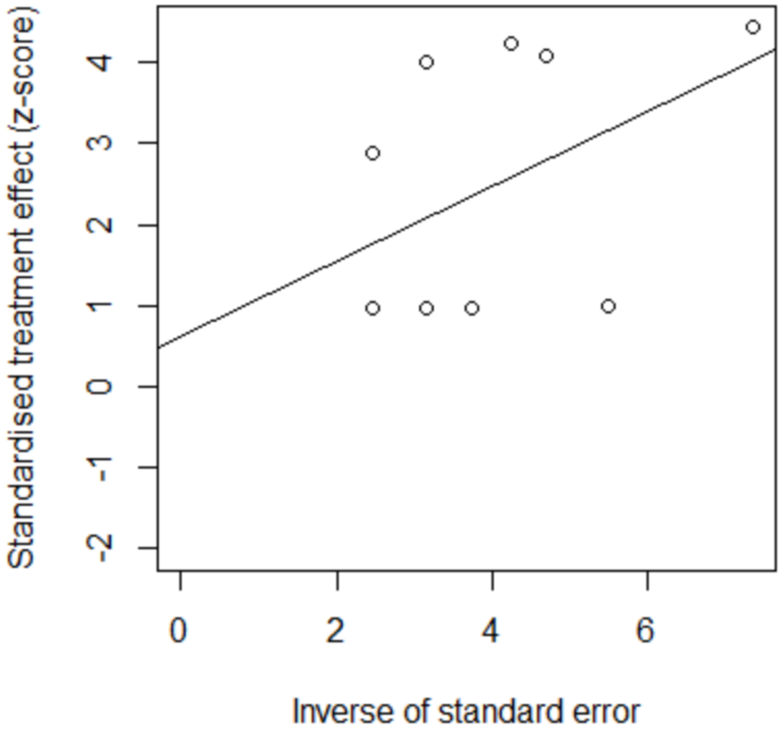

Leave one out forest plot for early mortality during Covid-19 pandemic

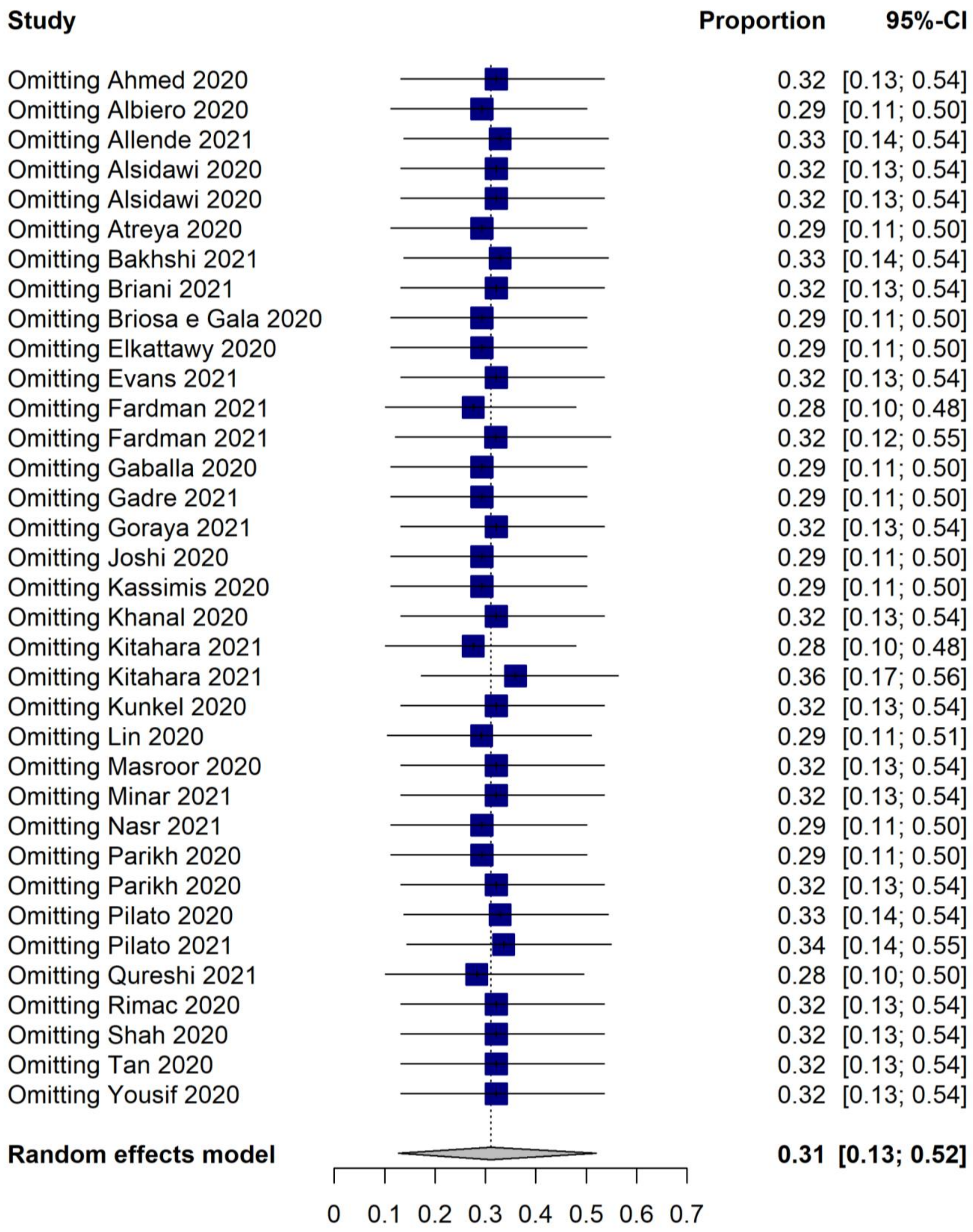

Pooled prevalence of early mortality after surgery before and during Covid-19 pandemic

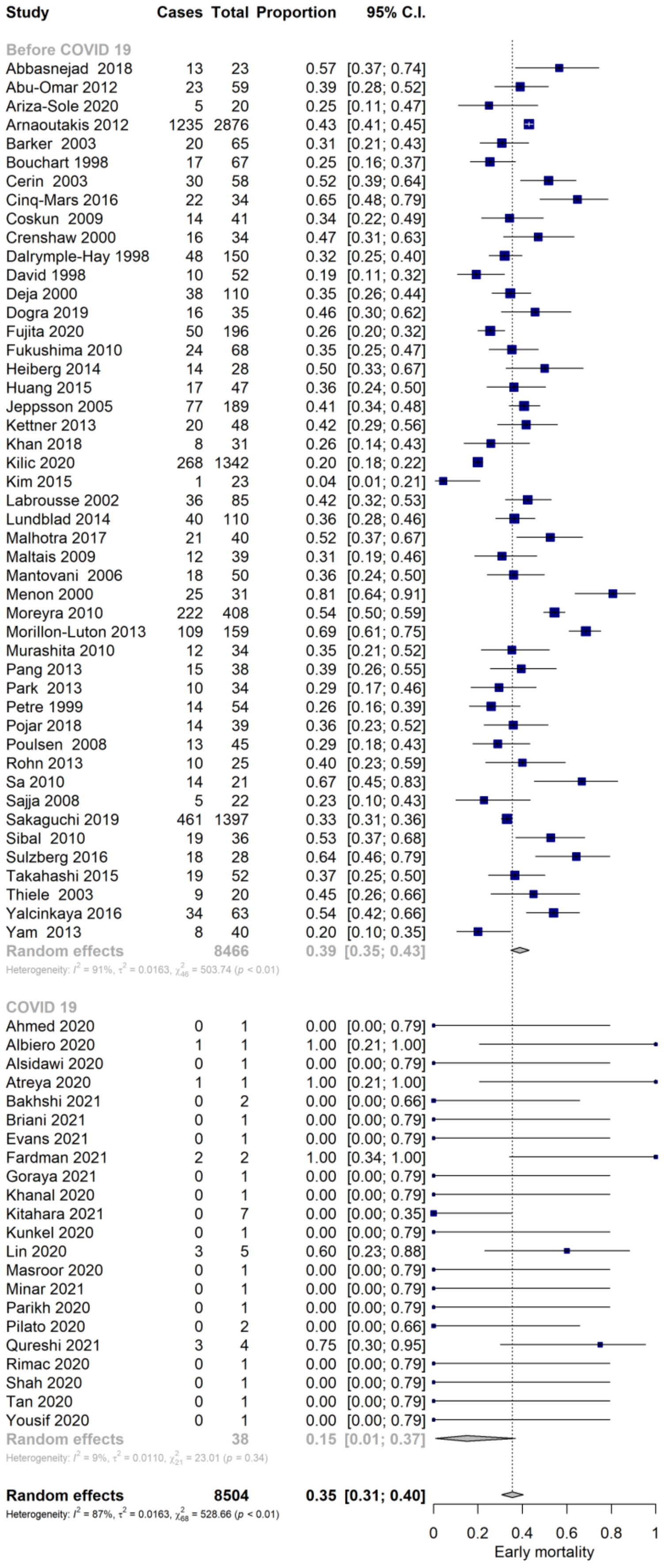

Funnel plot (A) and radial plot (B) for early mortality after surgery before and during Covid-19 pandemic showing no evidence of publication bias and heterogeneity among studies (Egger's test, P= 0.77), respectively.

A

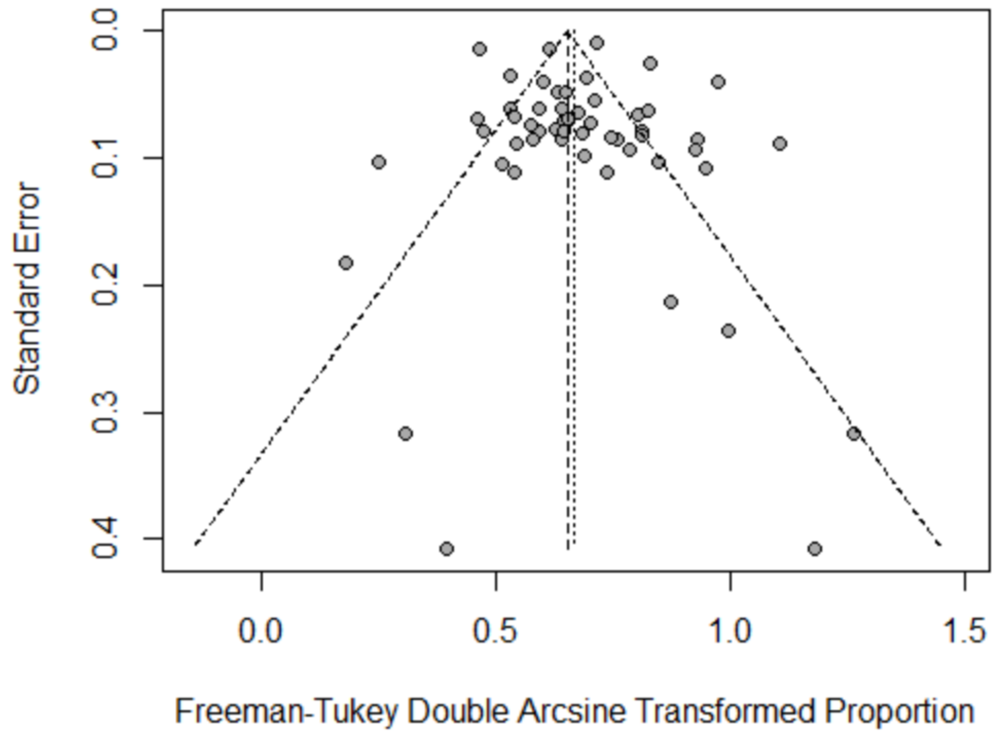

B

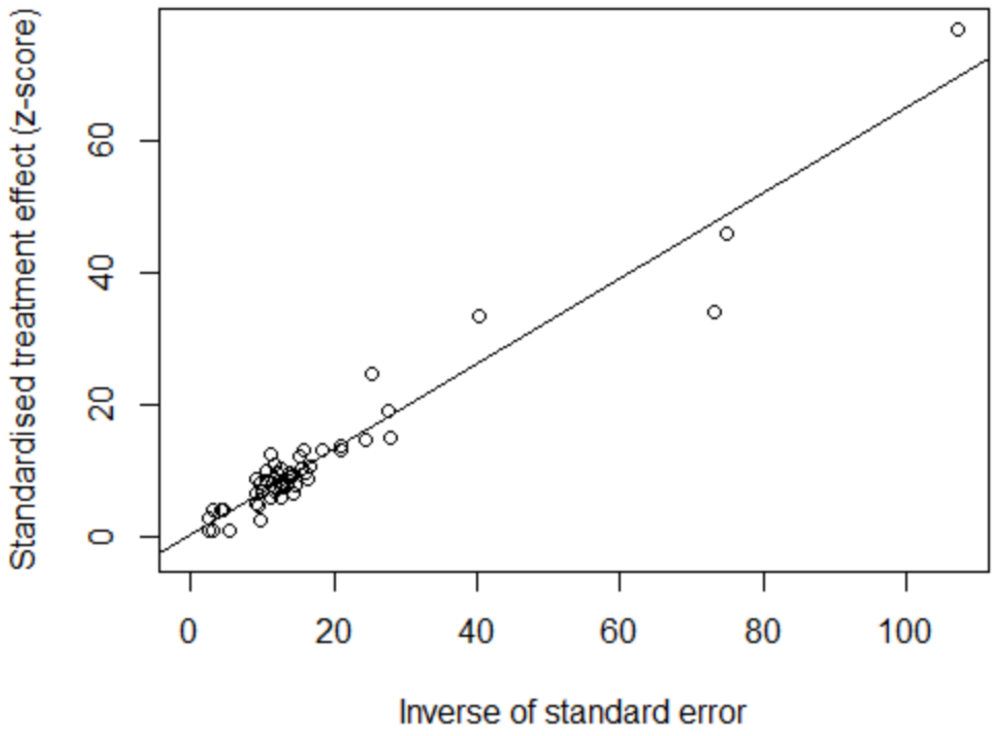

Pooled prevalence of early mortality after conservative treatment before and during Covid-19 pandemic

Study	Cases	Total	Proportion	95% C.I.
**No surgery - Before COVID 19**				
Abbasnejad 2018	40	41	0.98	[0.87; 1.00]
Ariza-Sole 2020	8	8	1.00	[0.68; 1.00]
Heiberg 2014	10	12	0.83	[0.55; 0.95]
Malhotra 2017	10	10	1.00	[0.72; 1.00]
Menon 2000	23	24	0.96	[0.80; 0.99]
Morillon-Luton 2013	59	64	0.92	[0.83; 0.97]
Poulsen 2008	19	19	1.00	[0.83; 1.00]
Sajja 2008	3	3	1.00	[0.44; 1.00]
Random effects		.	0.98	[0.94; 1.00]
Heterogeneity: $I^2 = 0\%$, $\tau^2 = 0$, $\chi^2_7 = 5.24$ ($p = 0.63$)				
**No surgery - COVID 19**				
Allende 2021	0	2	0.00	[0.00; 0.66]
Alsidawi 2020	0	1	0.00	[0.00; 0.79]
Briosa e Gala 2020	1	1	1.00	[0.21; 1.00]
Elkattawy 2020	1	1	1.00	[0.21; 1.00]
Fardman 2021	4	13	0.31	[0.13; 0.58]
Gaballa 2020	1	1	1.00	[0.21; 1.00]
Gadre 2021	1	1	1.00	[0.21; 1.00]
Joshi 2020	1	1	1.00	[0.21; 1.00]
Kassimis 2020	1	1	1.00	[0.21; 1.00]
Kitahara 2021	2	2	1.00	[0.34; 1.00]
Nasr 2021	1	1	1.00	[0.21; 1.00]
Parikh 2020	1	1	1.00	[0.21; 1.00]
Pilato 2021	0	3	0.00	[0.00; 0.56]
Random effects		.	0.62	[0.27; 0.93]
Heterogeneity: $I^2 = 35\%$, $\tau^2 = 0.0562$, $\chi^2_{12} = 18.54$ ($p = 0.10$)				
**Random effects**		.	**0.95**	**[0.80; 1.00]**
Heterogeneity: $I^2 = 67\%$, $\tau^2 = 0.0532$, $\chi^2_{20} = 59.74$ ($p < 0.01$)				

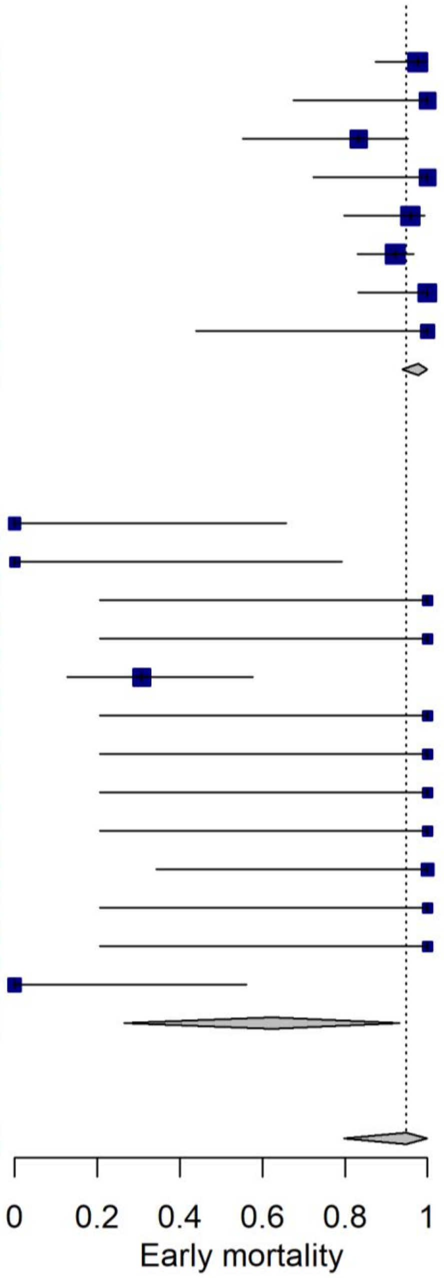

Funnel plot (A) and radial plot (B) for early mortality after conservative treatment before and during Covid-19 pandemic showing no evidence of publication bias and heterogeneity among studies (Egger's test, P= 0.06), respectively.

A

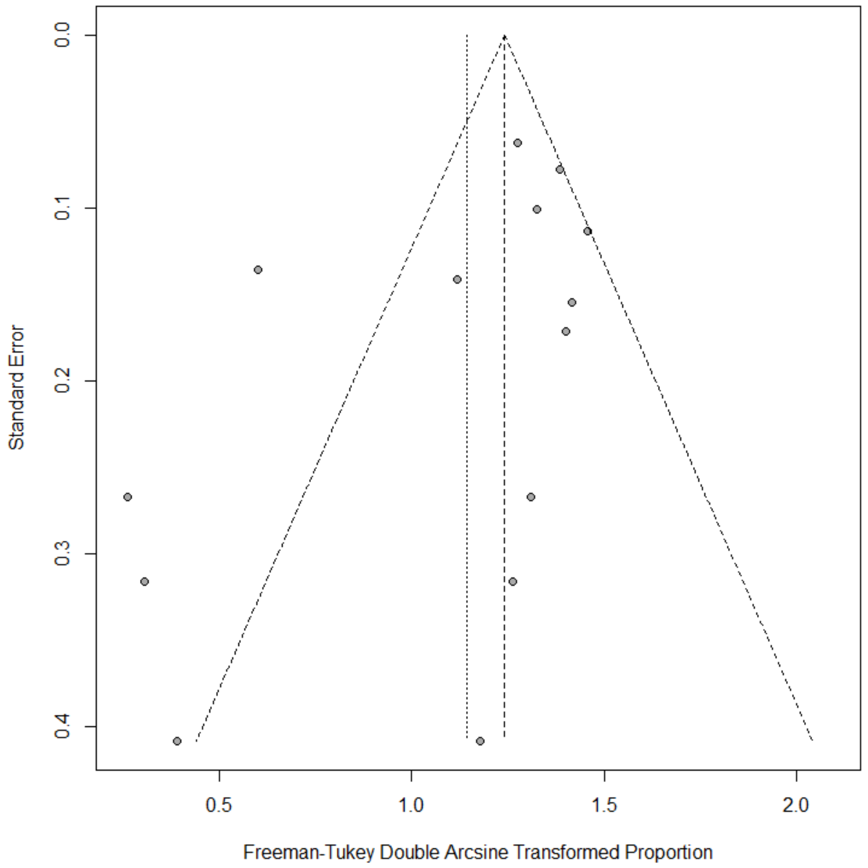

B

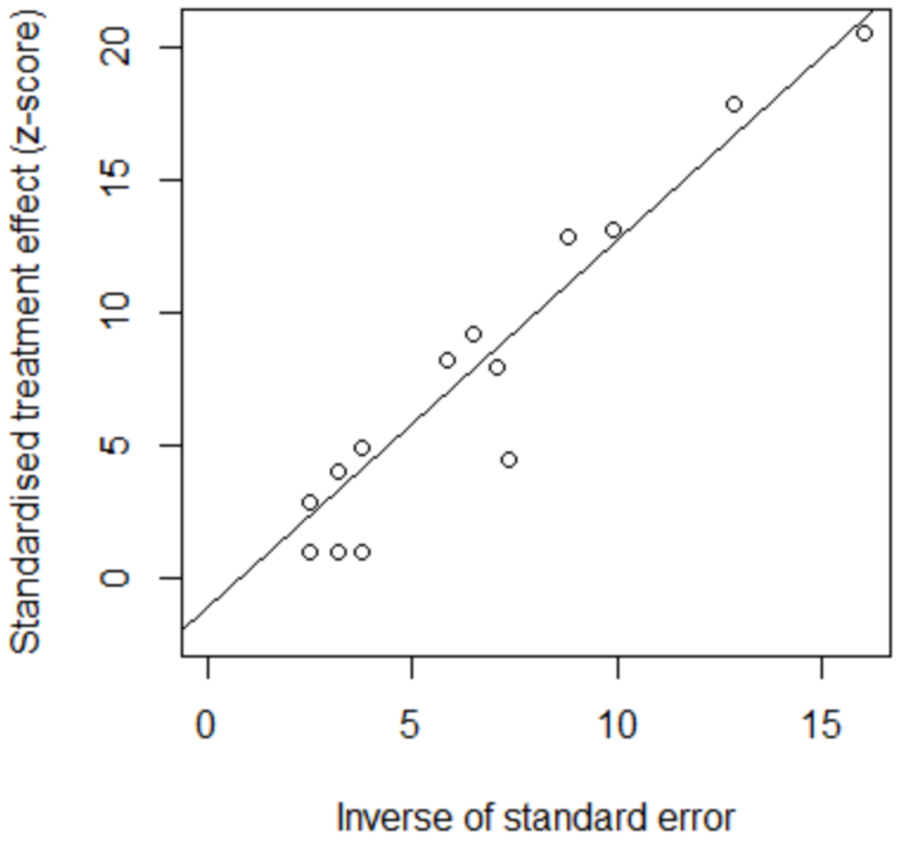

Results of multiple meta-regression analysis for early mortality.

Variable	Regression coefficient	Standard error	Lower 95% confidence interval	Upper 95% confidence interval	P Value
**Surgery**					
Year of publication*	0.000072	0.0032	-0.0063	0.0064	0.98
Covid-19 pandemic*	-0.12	0.090	-0.30	0.058	0.18
**Conservative treatment**					
Year of publication$^{\delta}$	0.00048	0.0091	-0.019	0.020	0.96
Covid-19 pandemic$^{\delta}$	-0.53	0.14	-0.82	-0.25	0.001

* p for interaction=0.88

$^{\delta}$ p for interaction=0.56

PRISMA checklist

Section and Topic	Item #	Checklist item	Location where item is reported
**TITLE**			
Title	1	Identify the report as a systematic review.	1
**ABSTRACT**			
Abstract	2	See the PRISMA 2020 for Abstracts checklist.	3
**INTRODUCTION**			
Rationale	3	Describe the rationale for the review in the context of existing knowledge.	5
Objectives	4	Provide an explicit statement of the objective(s) or question(s) the review addresses.	5
**METHODS**			
Eligibility criteria	5	Specify the inclusion and exclusion criteria for the review and how studies were grouped for the syntheses.	5 + SUPPL
Information sources	6	Specify all databases, registers, websites, organisations, reference lists and other sources searched or consulted to identify studies. Specify the date when each source was last searched or consulted.	5 + SUPPL
Search strategy	7	Present the full search strategies for all databases, registers and websites, including any filters and limits used.	5 + SUPPL
Selection process	8	Specify the methods used to decide whether a study met the inclusion criteria of the review, including how many reviewers screened each record and each report retrieved, whether they worked independently, and if applicable, details of automation tools used in the process.	5 + SUPPL
Data collection process	9	Specify the methods used to collect data from reports, including how many reviewers collected data from each report, whether they worked independently, any processes for obtaining or confirming data from study investigators, and if applicable, details of automation tools used in the process.	5 + SUPPL
Data items	10a	List and define all outcomes for which data were sought. Specify whether all results that were compatible with each outcome domain in each study were sought (e.g. for all measures, time points, analyses), and if not, the methods used to decide which results to collect.	5 + SUPPL
	10b	List and define all other variables for which data were sought (e.g. participant and intervention characteristics, funding sources). Describe any assumptions made about any missing or unclear information.	5 + SUPPL
Study risk of bias assessment	11	Specify the methods used to assess risk of bias in the included studies, including details of the tool(s) used, how many reviewers assessed each study and whether they worked independently, and if applicable, details of automation tools used in the process.	5 + SUPPL
Effect measures	12	Specify for each outcome the effect measure(s) (e.g. risk ratio, mean difference) used in the synthesis or presentation of results.	5 + SUPPL
Synthesis methods	13a	Describe the processes used to decide which studies were eligible for each synthesis (e.g. tabulating the study intervention characteristics and comparing against the planned groups for each synthesis (item #5)).	5 + SUPPL
	13b	Describe any methods required to prepare the data for presentation or synthesis, such as handling of missing summary statistics, or data conversions.	5 + SUPPL
	13c	Describe any methods used to tabulate or visually display results of individual studies and syntheses.	5 + SUPPL
	13d	Describe any methods used to synthesize results and provide a rationale for the choice(s). If meta-analysis was performed, describe the model(s), method(s) to identify the presence and extent of statistical heterogeneity, and software package(s) used.	5 + SUPPL
	13e	Describe any methods used to explore possible causes of heterogeneity among study results (e.g. subgroup analysis, meta-regression).	5 + SUPPL
	13f	Describe any sensitivity analyses conducted to assess robustness of the synthesized results.	5 + SUPPL
Reporting bias assessment	14	Describe any methods used to assess risk of bias due to missing results in a synthesis (arising from reporting biases).	5 + SUPPL
Certainty assessment	15	Describe any methods used to assess certainty (or confidence) in the body of evidence for an outcome.	5 + SUPPL
**RESULTS**			
Study selection	16a	Describe the results of the search and selection process, from the number of records identified in the search to the number of studies included in the review, ideally using a flow diagram.	6 + SUPPL
	16b	Cite studies that might appear to meet the inclusion criteria, but which were excluded, and explain why they were excluded.	6 + SUPPL
Study characteristics	17	Cite each included study and present its characteristics.	6 + SUPPL
Risk of bias in studies	18	Present assessments of risk of bias for each included study.	6 + SUPPL
Results of individual studies	19	For all outcomes, present, for each study: (a) summary statistics for each group (where appropriate) and (b) an effect estimate and its precision (e.g. confidence/credible interval), ideally using structured tables or plots.	6 + SUPPL
Results of	20a	For each synthesis, briefly summarise the characteristics and risk of bias among contributing studies.	6 + SUPPL

Section and Topic	Item #	Checklist item	Location where item is reported
syntheses	20b	Present results of all statistical syntheses conducted. If meta-analysis was done, present for each the summary estimate and its precision (e.g. confidence/credible interval) and measures of statistical heterogeneity. If comparing groups, describe the direction of the effect.	6 + SUPPL
	20c	Present results of all investigations of possible causes of heterogeneity among study results.	6 + SUPPL
	20d	Present results of all sensitivity analyses conducted to assess the robustness of the synthesized results.	6 + SUPPL
Reporting biases	21	Present assessments of risk of bias due to missing results (arising from reporting biases) for each synthesis assessed.	6 + SUPPL
Certainty of evidence	22	Present assessments of certainty (or confidence) in the body of evidence for each outcome assessed.	6 + SUPPL
**DISCUSSION**			
Discussion	23a	Provide a general interpretation of the results in the context of other evidence.	6
	23b	Discuss any limitations of the evidence included in the review.	7
	23c	Discuss any limitations of the review processes used.	7
	23d	Discuss implications of the results for practice, policy, and future research.	7
**OTHER INFORMATION**			
Registration and protocol	24a	Provide registration information for the review, including register name and registration number, or state that the review was not registered.	5 + SUPPL
	24b	Indicate where the review protocol can be accessed, or state that a protocol was not prepared.	5 + SUPPL
	24c	Describe and explain any amendments to information provided at registration or in the protocol.	5 + SUPPL
Support	25	Describe sources of financial or non-financial support for the review, and the role of the funders or sponsors in the review.	9
Competing interests	26	Declare any competing interests of review authors.	9
Availability of data, code and other materials	27	Report which of the following are publicly available and where they can be found: template data collection forms; data extracted from included studies; data used for all analyses; analytic code; any other materials used in the review.	5 + SUPPL

From: Page MJ, McKenzie JE, Bossuyt PM, Boutron I, Hoffmann TC, Mulrow CD, et al. The PRISMA 2020 statement: an updated guideline for reporting systematic reviews. BMJ 2021;372:n71. doi: 10.1136/bmj.n71
